# 360° Contextual Simulation Videos for Undergraduate Nursing Students: Electroencephalography-Based Quasi-Experimental Study

**DOI:** 10.2196/84720

**Published:** 2026-06-30

**Authors:** Yi-Chang Chen, Chen-Jung Chen

**Affiliations:** 1 Institute of Long-Term Care, MacKay Medical University, New Taipei City, Taiwan; 2 Department of Nursing, MacKay Medical University, No. 46, Sec. 3, Zhongzheng Rd., Sanzhi Dist., New Taipei City, 252, Taiwan, 886 2-26360303 ext 1325, 886 2-226361267

**Keywords:** computer, education, electroencephalography, EEG, learning, nursing, satisfaction, simulation, training, video

## Abstract

**Background:**

Screen-based 360° contextual simulation videos are increasingly being used in nursing education; however, evidence regarding their effects on learning outcomes and neural electroencephalography (EEG) patterns remains limited.

**Objective:**

This study aims to evaluate whether integrating 360° contextual simulation videos with traditional classroom instruction influences nursing students’ perceptions of simulation design and educational practices, their levels of satisfaction and self-confidence in learning, and EEG-based neural correlates of action observation.

**Methods:**

In a quasi-experiment, 55 third-year nursing students (experimental n=28; control n=27) were assigned to 360° contextual simulation video learning (laptop; no head-mounted display) or traditional classroom-based instruction (didactic instruction, guided discussion, and 2D instructional videos). The primary outcomes were Simulation Design Scale (SDS) and Educational Practices Questionnaire (EPQ) scores. Secondary outcomes included Student Satisfaction and Self-Confidence in Learning Scale (SCLS) scores and EEG indices (power at C3, Cz, and C4; μ-rhythm suppression scores). Group differences were tested with 2-tailed *t* tests and analysis of covariance adjusted for baseline values.

**Results:**

Between-group analyses (analysis of covariance adjusted for baseline values) revealed higher posttest scores on the SDS (*F*_1,35_=7.80, *η*²=.182; *P*=.008), EPQ (*F*_1,35_=6.00, *η*²=.146; *P*=.02), and SCLS (*F*_1,35_=7.01, *η*²=.167; *P*=.01) in the experimental group compared to the control group. Within-group analyses (paired *t* tests) indicated significant increases in SDS and EPQ scores for the experimental group (*P*<.001 and *P*=.005), whereas the control group showed a decrease in the SDS score (*P*=.001) and no significant change in the EPQ score (*P*=.11). The SCLS score did not significantly change in the experimental group (*P*=.21) but increased in the control group (*P*=.04). EEG analyses revealed significant postexposure reductions in power at C3, Cz, and C4 for the experimental group (all *P*<.001), with between-group effects at Cz (*F*_1,35_=425.29, *η*²=.926; *P*<.001) and C4 (*F*_1,35_=5.45, *η*²=.138; *P*=.03). μ-Suppression, which is an indirect EEG correlate that is often reported during action observation, was greater in the experimental group at Cz (*P*=.03) and C4 (*P*=.03) but not at C3 (*P*=.13). No adverse events were reported.

**Conclusions:**

Integrating 360° contextual simulation videos into undergraduate nursing education was associated with higher SDS and EPQ scores and improved self-reported satisfaction and self-confidence, in addition to EEG-based findings that may reflect neural EEG patterns associated with action observation. Larger multisite randomized trials are warranted to confirm efficacy and cost-effectiveness.

## Introduction

### Problem and Rationale

Simulation-based learning has emerged as an essential pedagogical approach in undergraduate nursing education, especially for skill-based disciplines, such as nursing [1,2]. In particular, simulation-based learning has significant value in resource-limited settings where clinical or practical training opportunities may be restricted [1,3]. In undergraduate nursing education, repeated supervised practice is often constrained by limited clinical placements and patient access, limited faculty supervision time, and the limited availability of high-fidelity simulation facilities. Therefore, simulation-based learning can provide a standardized and safe environment for repeated observation, practice preparation, and feedback to support foundational skill acquisition and clinical decision-making [1-4]. Contextual simulations replicate real-world scenarios in controlled learning environments, thus allowing students to develop critical skills in a safe and structured manner [2]. This approach has been widely recognized for its ability to improve learners’ knowledge, skills, and behavioral performance and to support clinical decision-making abilities in health professions education [1,4]. The effectiveness of simulation-based education has been well documented in nursing education and broader health professions education [1,4].

### What Is Known About Screen-Based 360° Contextual Simulation Videos

Screen-based 360° contextual simulation videos (laptop, no head-mounted display) can provide standardized exposure to nursing procedures and clinical contexts while allowing learners to control their viewing direction during action observation, which is particularly relevant for early-stage skill acquisition and preparation before supervised practice [5,6]. With recent advancements in more immersive panoramic viewing experience learning technologies, screen-based 360° contextual simulation videos have been identified as innovative alternatives to conventional instructional methods across undergraduate nursing education contexts [5,6]. Recent systematic reviews and meta-analyses have also supported the effectiveness of immersive virtual reality in nursing education for improving learners’ knowledge, skills, and problem-solving abilities [7]. Unlike standard fixed-view videos, our screen-based 360° contextual simulation video system allowed learners to actively control their viewing direction while observing context-rich clinical procedures on a laptop screen without a head-mounted display [5,6].

### Knowledge Gap

Despite the increasingly widespread adoption of screen-based 360° contextual simulation videos in undergraduate nursing skills training, empirical evidence remains limited, particularly in resource-limited educational settings where supervised practice opportunities are constrained by limitations with respect to clinical patient access, faculty time, and access to high-fidelity simulation facilities [3]. In this context, evidence of the efficacy of screen-based 360° contextual simulation videos—especially regarding observational learning during procedural skill observation and associated electroencephalography (EEG)-based indices during action observation (eg, μ-rhythm suppression)—remains sparse [6,8]. Existing studies have rarely examined both (1) learners’ perceptions of simulation quality and educational practices and (2) learners’ satisfaction and self-confidence alongside EEG-based indices during action observation [6]. Clarifying these outcomes together may help educators understand not only how students perceive screen-based 360° contextual simulation videos but also how such exposure relates to EEG patterns that are commonly reported during procedural observation. However, our understanding of the direct impact of screen-based 360° contextual simulation videos on cognitive and neural mechanisms, particularly with respect to skill acquisition, learning retention, and neurophysiological engagement, remains limited [6,9]. In addition, the use of EEG to analyze neural correlates in contexts involving screen-based 360° contextual simulation video learning remains relatively underexplored [9]. Accordingly, this study addresses this gap using a 2-group comparison to examine students’ perceived simulation design and educational practices, satisfaction and self-confidence in learning, and sensorimotor EEG indices during procedural action observation in response to a screen-based 360° contextual simulation video intervention. Furthermore, investigations into how these simulations influence neural engagement, particularly through observational learning processes, have been limited.

### Study Justification and Aim

Mirror neuron theory proposes that observing others’ actions can engage the neural systems involved in action execution, supporting observational learning [10]. In EEG research, μ-rhythm suppression over sensorimotor regions is commonly reported during action observation and is often interpreted as an indirect neural correlate that is consistent with mirror neuron system engagement; however, it is not a direct measure of mirror neuron EEG patterns [8,11]. This framework offers a plausible neurophysiological explanation, suggesting that observing tasks in contextual settings stimulates neural circuits that are associated with action execution and cognitive processing and may support motor skill acquisition and memory retention [8,10]. Specifically, EEG studies have identified μ-rhythm suppression as an indirect marker that is consistent with mirror neuron system engagement in the context of observational learning within immersive environments [8,11]. The Kolb experiential learning framework was used as a guiding lens through which to interpret how video-based exposure and facilitated reflection may support early phases of skill learning in undergraduate nursing education [12]. Additionally, recent neuroeducation research suggests that observational learning in immersive media may engage the mirror neuron system, as indexed by EEG μ-rhythm suppression [8,11]. Furthermore, cognitive load theory provides a useful design lens for screen-based simulation by emphasizing strategies that reduce extraneous load and support meaningful processing during procedural learning [13].

To our knowledge, few studies have directly integrated EEG-based learning analytics with screen-based 360° contextual simulation videos in undergraduate education. As multimodal learner data—including biometric signals—are increasingly leveraged in artificial intelligence–enhanced learning analytics to enable real-time feedback and personalization, understanding how immersive simulations influence neural engagement is important for informing adaptive educational models [14]. The aim of this study was to evaluate the effects of screen-based 360° contextual simulation videos on undergraduate nursing students’ (1) perceived simulation design and educational practices, (2) satisfaction and self-confidence in learning, and (3) sensorimotor EEG indices during procedural action observation (sensorimotor power and μ-rhythm suppression) by comparing an experimental group with a control group using questionnaires and EEG measures. The screen-based 360° contextual simulation videos were delivered on a laptop screen without a head-mounted display; therefore, the intervention is distinct from headset-based virtual reality simulation.

## Methods

### Design

This study had a 2-group, pretest-posttest quasi-experimental design with 2 assessment time points. The participants were assigned to either an experimental group or a control group using a computer-generated allocation sequence. However, because recruitment was convenience-based within a single university cohort and blinding was not implemented, the study is reported as quasi-experimental. The reporting of this study was guided by the CONSORT (Consolidated Standards of Reporting Trials) 2010 checklist, where applicable (Checklist 1).

### Setting and Participants

Participants were recruited through announcements made in undergraduate courses and departmental emails within a university setting, and a nursing cohort was used as the case sample. An a priori power analysis was conducted using G*Power to estimate the minimum sample size planned for an analysis of covariance (ANCOVA) framework (2 groups, 1 covariate), assuming a medium effect size (*f*=0.25) in the absence of robust preliminary data, with *α*=.05 and power=0.80, yielding a minimum required sample of 34. The inclusion criteria for this study were as follows: (1) being a third-year undergraduate student in a nursing program, (2) being able to converse in Taiwanese and Mandarin, and (3) being willing to participate voluntarily in the research. The exclusion criteria were deafness or cognitive impairment according to various sources (such as school health information and parental notification). The participants were assigned to either the experimental group (screen-based 360° contextual simulation video learning) or the control group (dose-matched screen-based 2D instructional video learning), with the video learning conducted during after-class skills practice sessions.

### Procedures

The study was conducted over 1 week in July 2024 and included 2 assessment time points: baseline (T0) and immediately postintervention (T1). On day 1, all participants completed baseline questionnaires and underwent baseline EEG recording. Pretest EEG recording was conducted in the eyes-open resting condition and did not involve viewing any simulation content or videos. After the baseline assessment, the participants were allocated to either the experimental or control group. Both groups completed 4 learning modules within the week. Each module followed the same overall structure in both groups: a brief orientation, video viewing on a laptop (approximately 10‐12 min), and a 20-minute instructor-facilitated discussion using structured prompts; each module was designed to be completed within approximately 60 minutes. Immediately after the final module, posttest questionnaires were administered and collected under standardized proctoring by faculty members and teaching assistants, and the participants underwent posttest EEG recording. This procedure was identical for the control group; the posttest EEG data were recorded immediately after the fourth control module in the same eyes-open resting condition.

### Theoretical Framework

As noted in the *Introduction* section, the Kolb experiential learning cycle informed the design and interpretation of the intervention; the operationalization of each stage in this study is described below. Concrete experience was operationalized as students’ exposure to screen-based 360° procedural scenarios. Reflective observation was supported through structured, instructor-guided debriefing immediately after viewing. Abstract conceptualization was supported by linking the observed actions to key procedural principles and checklists during the discussion and through assigned review materials. Active experimentation was operationalized as students’ subsequent application of the observed steps during routine skills practice (outside the 360° viewing session) using the same procedural checklist and course materials [12].

### Interventions

The study included 2 intervention conditions delivered via the university’s institutional eLearning platform and accessed through a standard web browser on laptop computers. The 4 modules covered (1) the collection of urine specimens from a urine drainage bag, (2) the measurement and documentation of vital signs, (3) infant and pediatric cardiopulmonary resuscitation, and (4) chest physiotherapy. The modules were matched by topic and intended duration across groups and were completed during after-class skills practice sessions within the 1-week study period.

### Experimental Condition: Screen-Based 360° Contextual Simulation Videos

The experimental intervention consisted of screen-based 360° contextual simulation video learning delivered on laptop computers without a head-mounted display. In this study, “immersion” refers to panoramic 360° first-person viewing of realistic clinical procedures with context-rich audiovisual cues, and “interactivity” refers to learners’ ability to actively control their viewing direction (through panning and rotating) during observation. Because the videos were presented on a laptop screen, the level of immersion and interaction was lower than that of fully immersive headset-based virtual reality. All screen-based 360° contextual simulation videos were self-developed by the nursing faculty and information engineering professionals through interdisciplinary discussions and were delivered via the institutional eLearning platform. Before each module, the students received a brief orientation that included learning objectives, instructions on how to navigate the 360° video (eg, how to change viewing direction), and expectations for participation. Each module included viewing of the corresponding screen-based 360° contextual simulation video (approximately 10‐12 min), followed by a 20-minute facilitated contextual debriefing led by faculty members using structured prompts focused on key steps, safety considerations, and clinical decision-making. Each module was designed to be completed within approximately 60 minutes and focused on a single, discrete nursing skill. The participants had no prior exposure to the 4 study-specific 360° modules before baseline assessment.

### Control Condition: Dose-Matched 2D Instructional Videos

The control group covered the same 4 skill topics and used dose-matched, screen-based 2D instructional videos and the same overall module structure and scheduling. The 2D instructional videos were developed by the same teaching team and delivered through the same institutional eLearning platform accessed on a laptop via a web browser. Before each module, the students received a brief orientation that included learning objectives, instructions on how to access and view the 2D video (fixed-view, with no panning or rotation control), and expectations for participation. The educational content included (1) didactic instruction and demonstration of key procedural steps, (2) viewing of the corresponding 2D instructional video (approximately 10‐12 min), and (3) a 20-minute instructor-facilitated discussion using the same structured prompts as the experimental condition (key steps, safety considerations, and clinical decision-making). Each module was designed to be completed within approximately 60 minutes and was matched to the experimental condition by topic and duration. The control activities did not involve high-fidelity mannequin simulation, standardized-patient scenarios, or 360° video viewing.

### Instructional Design and Standardization (Cognitive Load-Informed Features)

The intervention was designed with cognitive load theory–informed considerations to minimize extraneous demands and support learning efficiency. Across both conditions, each video was kept short (approximately 10‐12 min) and focused on a single skill topic. Key procedural steps were standardized using step-by-step procedural checklists reinforced during the instructor-guided discussion, and the learning environment and delivery platform were kept consistent across groups. We did not directly measure cognitive load; therefore, these elements are reported as design features rather than demonstrated mechanisms. Accordingly, unmeasured cognitive load and related factors (eg, attention or fatigue) may have influenced EEG indices and learning outcomes, which should be interpreted cautiously.

### Questionnaire Measures

Quantitative data were collected via 1 demographic questionnaire and 3 additional questionnaires, and perceptions of simulation design and educational practices were assessed using the Simulation Design Scale (SDS) and the Educational Practices Questionnaire (EPQ), respectively. Satisfaction and self-confidence in learning were assessed using the Student Satisfaction and Self-Confidence in Learning Scale (SCLS). All of these instruments are contextual simulation research questionnaires developed by the National League for Nursing [15]. The reliability of the questionnaires was evaluated according to Cronbach α coefficient. Experts have recommended the use of these measurement questionnaires when implementing contextual simulations in education programs [15].

The SDS is composed of 20 items and is used to assess students’ views concerning design elements pertaining to simulation and their importance. The questions address course themes and knowledge, support, problem-solving, feedback, and simulation contexts. In terms of reliability, the Cronbach α coefficient for the overall SDS was 0.96. The α coefficients for the knowledge of course themes, support, problem-solving, feedback, and simulation context scales were 0.92, 0.92, 0.86, 0.90, and 0.87, respectively. In terms of validity, the root mean square error of approximation (RMSEA), normed fit index (NFI), weighted root mean square residual (WRMR), and chi-square significance tests indicated a poor fit, whereas the comparative fit index (CFI), Tucker-Lewis index (TLI), and adjusted goodness-of-fit index (AGFI) values were greater than the commonly accepted thresholds. The conceptual model accounted for 17 out of 20 SDS items (85%). The correlations between different pairs of theoretical factors ranged between 0.67 and 0.89 [16].

The EPQ comprises 16 questions and is used to evaluate students’ views concerning the importance of using simulations in virtual environments in educational practice. The responses are scored on a 5-point Likert-type scale. These 16 items cover various subscales, including active learning, cooperation, varied learning styles, and high expectations. In terms of reliability, the Cronbach α coefficient for the overall EPQ was 0.95. The α coefficients for the active learning, cooperation, diversified learning style, and high expectation subscales were 0.93, 0.90, 0.88, and 0.88, respectively. In terms of validity, the RMSEA, WRMR, and chi-square significance tests indicated that the fit was not good, whereas the CFI, TLI, NFI, and AGFI values were greater than the commonly acceptable thresholds. The conceptual model accounted for 13 out of 16 EPQ items (approximately 80%). The correlations between different pairs of conceptual factors ranged between 0.77 and 0.86 [16].

The SCLS comprises 14 items and is used to measure participants’ satisfaction with and confidence in the current learning protocol on a 5-point Likert-type scale. In terms of reliability, the Cronbach α coefficient for the entire SCLS was 0.92, and the α coefficients for the satisfaction and confidence subscales were 0.92 and 0.83, respectively. In terms of validity, the RMSEA, WRMR, and chi-square significance tests indicated that the degree of fit was not good, whereas the CFI, TLI, NFI, and AGFI values were greater than the commonly accepted thresholds. The conceptual model accounted for 11 out of 14 (approximately 76%) SCLS items. The correlation coefficient between the satisfaction and confidence factors was 0.78 [16].

### EEG Measures

**Figure 1.**
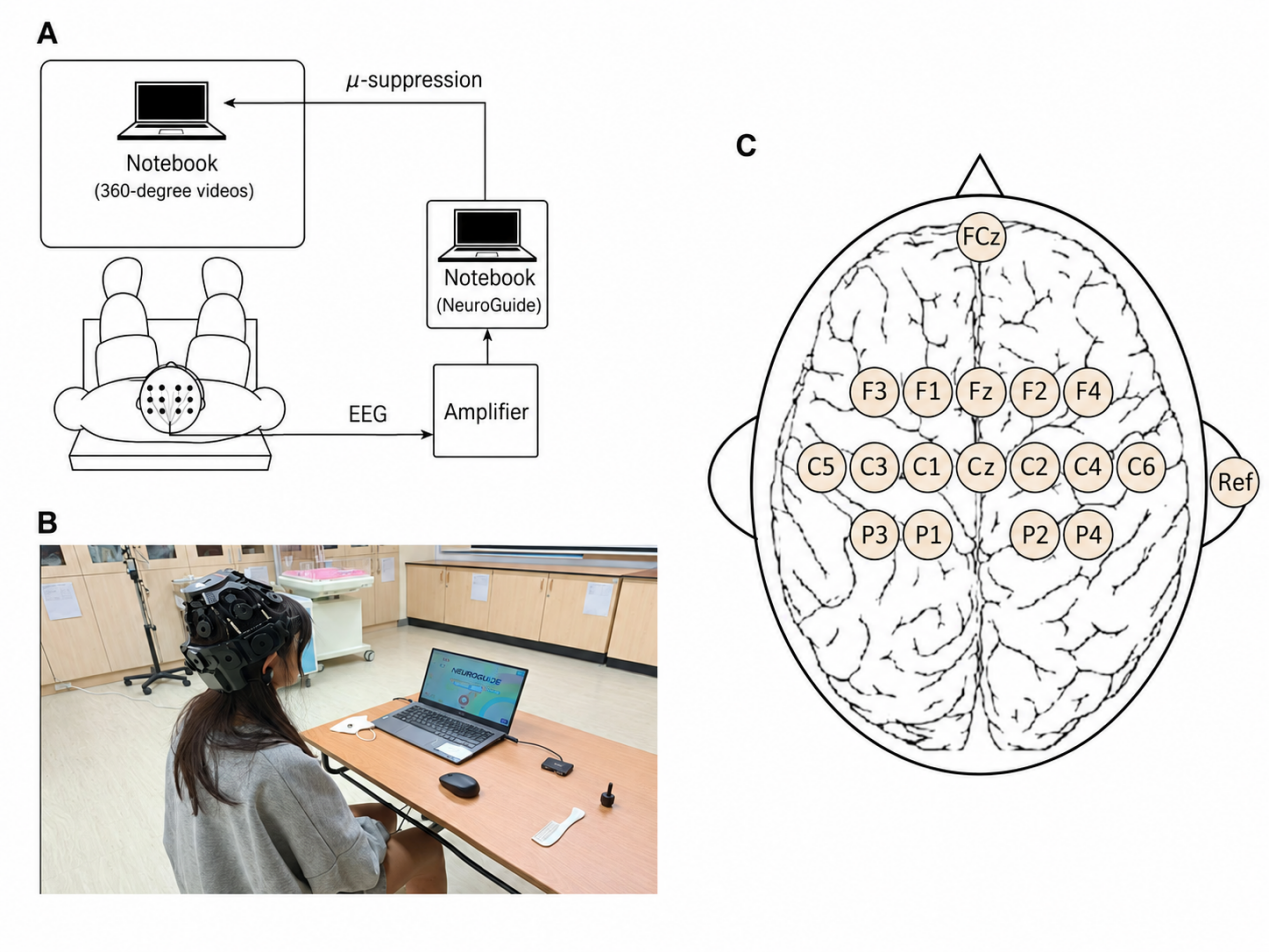
Schematic representation of the experimental setup for electroencephalography (EEG)-based analysis in pediatric nursing training (**A**), a participant using the screen-based 360° contextual simulation video system with EEG recording (**B**), and electrode placement for EEG data acquisition (**C**).

EEG recordings were obtained in a quiet room with the participants seated comfortably in front of a laptop screen. Electrode impedances were checked before each recording and maintained below the manufacturer-recommended threshold throughout the session. The baseline EEG (T0) data were recorded under standardized resting conditions prior to any module exposure, and the postintervention EEG (T1) data were recorded immediately after the completion of the module under the same recording setup. The same acquisition settings and recording environment were used for both groups to ensure comparability across time points and conditions. After the EEG recordings were obtained, the EEG data were analyzed by a professional pediatric neurologist based on a standardized protocol. The EEG system consisted of both hardware (DSI-24 EEG headset; Wearable Sensing) and software (NeuroGuide version 3.4.0; Applied Neuroscience, Inc.). The EEG data were collected with the assistance of a Neuroscan SynAmps system with a 0.1‐ to 30-Hz bandpass filter and a sampling rate of 500 Hz. For analysis, we extracted the first 160 seconds of eyes-open EEG data from each recording. The participants did not perform an active task during this segment (Figure 1). The μ waves were defined as the oscillations measured in the sensorimotor cortex; therefore, only the data obtained from C3, Cz, and C4 were analyzed. The EEG data were analyzed with the assistance of MATLAB and EEGLAB. The fast Fourier transform was applied with a Hanning window, and the power spectral density was computed for each 2-second epoch [17] (details are provided in Multimedia Appendix 1).

### Statistical Analysis

Missing questionnaire data were handled using a prespecified rule. The SDS, EPQ, and SCLS comprise 50 (20+16+14) items in total. Questionnaire records with ≥10 missing items at a given assessment time point were excluded from the questionnaire-based analyses because this level represents ≥20% missingness, a level at which scale scores may be unstable. All questionnaire- and EEG-derived outcomes were analyzed by an analyst who was not involved in intervention delivery or questionnaire administration [18] using IBM SPSS Statistics (version 25). Descriptive statistics are reported as the means (SDs). All statistical tests were 2-tailed, and statistical significance was set at *P*<.05. For the questionnaire outcomes, Pearson correlation coefficients were calculated to examine the associations between the pretest and posttest scores within each group. The analyses of the SDS and EPQ scores were treated as primary, whereas those of the SCLS scores and EEG indices were treated as secondary. Within-group pretest-to-posttest changes were evaluated using 2-tailed paired-samples *t* tests for each questionnaire outcome. Between-group differences in questionnaire outcomes were examined using ANCOVA, with the posttest score as the dependent variable, the group as the fixed factor, and the baseline (pretest) score as the covariate. Assumptions were assessed using Levene tests for homogeneity of variances and by testing the interaction between the group and baseline scores to evaluate the homogeneity of regression slopes. Effect sizes are reported as partial *η*² [19]. For EEG power measures at C3, Cz, and C4, Pearson correlation coefficients were calculated to examine the associations between the pretest and posttest values within each group. Within-group pretest-to-posttest changes in EEG power were evaluated using 2-tailed paired-samples *t* tests at each electrode site. Between-group differences in EEG power were assessed using Levene tests for homogeneity of variances and tests of the group × baseline interaction to evaluate the homogeneity of regression slopes. When the assumption of homogeneity of regression slopes was met, ANCOVA was conducted with the posttest EEG value as the dependent variable, the group as the fixed factor, and the baseline EEG value as the covariate. When the assumption was violated, ANCOVA was not performed for that electrode. Effect sizes are reported as partial *η*² [19]. Between-group differences in the μ-suppression scores at C3, Cz, and C4 were examined using 2-tailed independent-samples *t* tests. Levene tests were used to assess the homogeneity of variances prior to each *t* test.

### Ethical Considerations

The study protocol was conducted in accordance with institutional and national research ethics standards and the Declaration of Helsinki and approved by the Institutional Review Board of the MacKay Memorial Hospital, Taiwan (No. 21MMHIS438e). Informed consent was obtained from all participants. The participants were provided with a consent form on day 1 before baseline (T0), and consent was obtained immediately prior to administering the baseline questionnaires and conducting EEG recording. No participants withdrew after providing their consent. Participant privacy and confidentiality were protected throughout the study. All questionnaire and EEG data were coded and deidentified before analysis, and no names, student identification numbers, or other directly identifying information were included in the analytic dataset. Research data were stored in password-protected files and were accessible only to authorized members of the research team. The findings are reported in aggregate form only. Participants each received NT $300 (US $9.5) as compensation for their time. A detailed study protocol is provided in Multimedia Appendix 2.

## Results

### Participants

**Figure 2.**
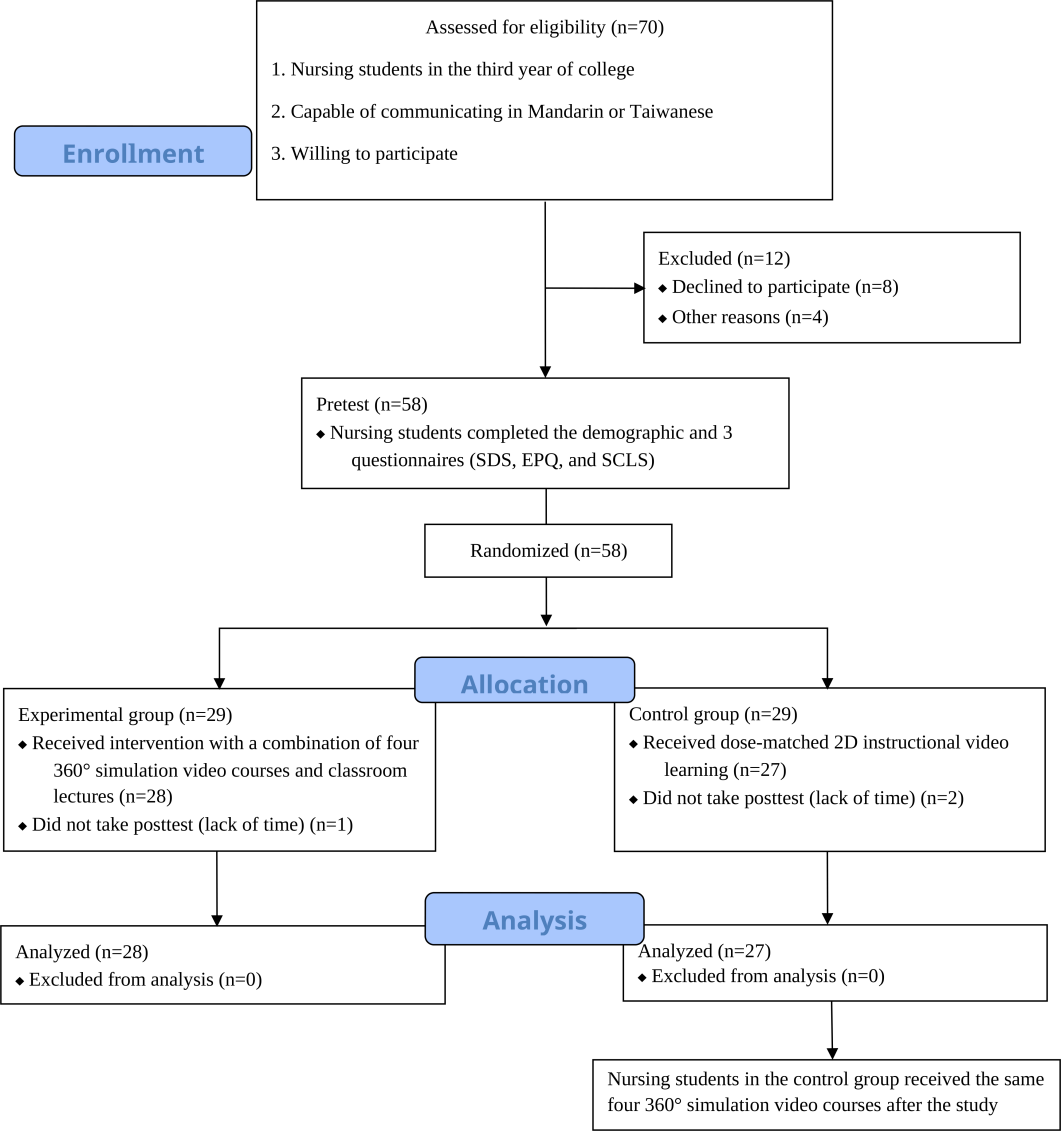
CONSORT (Consolidated Standards of Reporting Trials) flow diagram of participant recruitment, allocation, intervention, and analysis. EEG: electroencephalography; EPQ: Educational Practices Questionnaire; SCLS: Student Satisfaction and Self-Confidence in Learning Scale; SDS: Simulation Design Scale.

No questionnaire records were excluded at baseline (T0). At posttest (T1), 3 questionnaire records were excluded because of ≥10 missing items (1 in the experimental group and 2 in the control group). A total of 55 out of 70 students (79%), including 28 students in the experimental group and 27 students in the control group, completed both the pretest and posttest assessments. The CONSORT flow diagram, which outlines the participant recruitment and retention process used in this study, is shown in Figure 2. Baseline demographic characteristics and pretest outcome measures were comparable between the experimental and control groups, as shown in Table 1.

**Table 1.** Descriptive characteristics and group comparisons of the SDS^a^, EPQ^b^, SCLS^c^ scores, and EEG^d^ power measures.

Characteristic	Experimental group (n=28)	Control group (n=27)	Total (n=55)	Pearson *χ*^2^ (*df*)	*t* test (*df*)	*P* value	95% CI
Gender, n (%)				0.189 (1)	—^e^	.66	—
Male	7 (25)	8 (30)	15 (27)				
Female	21 (75)	19 (70)	40 (73)				
Academic performance (GPA^f^), n (%)				1.413 (3)	—	.70	—
2.6‐4.0	10 (36)	9 (33)	19 (35)				
1.6‐2.5	4 (14)	8 (30)	12 (22)				
1.1‐1.5	9 (32)	5 (19)	14 (25)				
0‐1.0	5 (18)	5 (19)	10 (18)				
Age (y), mean (SD)	16.85 (0.69)	17.00 (0.83)	16.95 (0.78)	—	0.577 (53)	.57	−0.39 to 0.69
Number of hours spent on the internet weekly, mean (SD)	37.15 (10.14)	37.76 (20.49)	37.56 (17.65)	—	0.125 (53)	.90	−9.20 to 10.41
SDS score, mean (SD)	77.54 (8.66)	75.26 (6.51)	76.00 (7.24)	—	−0.931 (53)	.36	−7.24 to 2.68
EPQ score, mean (SD)	64.46 (8.34)	59.81 (7.66)	61.33 (8.09)	—	−1.756 (53)	.09	−10.03 to 0.74
SCLS score, mean (SD)	61.77 (10.72)	58.04 (6.40)	59.25 (8.11)	—	−1.379 (53)	.18	−9.21 to 1.75
EEG power, mean (SD)							
C3	11.07 (6.9)	13.47 (11.85)	11.87 (8.80)	—	−0.797 (53)	.43	−8.48 to 3.69
Cz	40.60 (133.13)	71.20 (187.27)	50.80 (151.49)	—	−0.590 (53)	.56	−135.77 to 74.57
C4	14.24 (11.88)	15.84 (17.76)	14.78 (13.90)	—	−0.335 (53)	.74	−11.27 to 8.08

a SDS: Simulation Design Scale.

b EPQ: Educational Practices Questionnaire.

c SCLS: Student Satisfaction and Self-Confidence in Learning Scale.

d EEG: electroencephalography.

e Not applicable.

f GPA: grade point average, used as an indicator of academic performance [20].

### Effect of Screen-Based 360° Contextual Simulation Videos on Questionnaire Measures

#### Within-Group Analyses

In the experimental group, the correlation coefficients between the pretest and posttest scores for the SDS, EPQ, and SCLS were 0.258, −0.006, and −0.145, respectively. Two-tailed paired-sample *t* tests revealed statistically significant increases in the SDS (*t*_27_=−6.44; *P*<.001) and EPQ (*t*_27_=−3.47; *P*=.005) scores, whereas the changes observed in the SCLS scores were not statistically significant (*t*_27_=−1.32; *P*=.21; Table 2).

**Table 2.** Effects of the screen-based 360° contextual simulation videos on the SDS^a^, EPQ^b^, SCLS^c^ scores, and EEG^d^ power measures in the experimental and control groups (n=55).

Variable	Experimental group (n=28)			95% CI	Control group (n=27)			95% CI	ANCOVA term	*P* value_3_	*η* ^2^
	Mean (SD)	*t* test (*df*)	*P* value_1_		Mean (SD)	*t* test (*df*)	*P* value_2_				
SDS		–6.44 (27)	<.001^e^	–22.34 to –11.04		–5.50 (26)	.001^e^	–16.38 to –7.47			
Pretest	77.54 (8.66)				75.26 (6.51)				Covariate	.79	.002
Posttest	94.23 (6.41)				87.19 (8.03)				Group	.008^e^	.182
EPQ		–3.47 (27)	.005^e^	–16.90 to –3.87		–1.67 (26)	.11	–9.82 to 1.01			
Pretest	64.46 (8.34)				59.81 (7.66)				Covariate	.047^e^	.108
Posttest	74.85 (6.78)				64.22 (15.15)				Group	.02^e^	.146
SCLS		–1.32 (27)	.21	–11.44 to 2.81		–2.13 (26)	.04^e^	–5.90 to –0.10			
Pretest	61.77 (10.72)				58.04 (6.40)				Covariate	.45	.016
Posttest	66.08 (3.57)				61.04 (6.20)				Group	.01^e^	.167
C3^f^		3.93 (27)	<.001^e^	2.14 to 6.82		1.79 (26)	.10	–0.43 to 4.36			
Pretest	11.07 (6.95)				13.47 (11.85)				Covariate	—^g^	—
Posttest	6.60 (3.58)				11.50 (9.59)				Group	—	—
Cz^f^		5.41 (27)	<.001^e^	4.93 to 10.99		2.05 (26)	.06	–0.31 to 10.16			
Pretest	40.60 (133.13)				71.20 (187.27)				Covariate	<.001^e^	.998
Posttest	32.65 (131.11)				66.28 (183.41)				Group	<.001^e^	.926
C4^f^		4.82 (27)	<.001^e^	3.14 to 7.84		0.847 (26)	.41	–3.99 to 9.07			
Pretest	14.24 (11.88)				15.84 (17.76)				Covariate	<.001^e^	.698
Posttest	8.75 (10.05)				13.39 (12.08)				Group	.03^e^	.138

a SDS: Simulation Design Scale.

b EPQ: Educational Practices Questionnaire.

c SCLS: Student Satisfaction and Self-Confidence in Learning Scale.

d EEG: electroencephalography.

e *P*<.05. *P*_1_ was calculated by performing 2-tailed paired *t* tests within the experimental group, and *P*_2_ was calculated by performing 2-tailed paired *t* tests within the control group. *P*_3_ and *η*² were calculated by analysis of covariance to compare the posttest scores between the groups, and adjustments were made for baseline differences.

f C3, Cz, and C4: EEG electrode placement locations based on the international 10-20 system.

g Not applicable.

In the control group, the correlation coefficients between the pretest and posttest scores for the SDS, EPQ, and SCLS were −0.192, 0.435, and 0.323, respectively. Two-tailed paired-samples *t* tests indicated a statistically significant decrease in the SDS score (*t*_26_=−5.50; *P*=.001) and a statistically significant increase in the SCLS score (*t*_26_=−2.13; *P*=.04), whereas the change in the EPQ score (*t*_26_=−1.67; *P*=.11) was not statistically significant (Table 2).

#### Between-Group Analyses

The assumptions for ANCOVA were checked (homogeneity of variances and homogeneity of regression slopes) and were met for the SDS, EPQ, and SCLS scores (details are provided in Multimedia Appendix 3). ANCOVA revealed significant between-group effects for the SDS, EPQ, and SCLS scores (Table 2), indicating that exposure to the screen-based 360° contextual simulation videos significantly influenced these questionnaire outcomes. Effect sizes (partial *η*²) are reported in Table 2. Taken together, the results indicate that, relative to the dose-matched control condition, the 360° video condition was associated with more favorable posttest student-reported perceptions of simulation design and educational practices (SDS and EPQ) and higher satisfaction or self-confidence (SCLS) after adjusting for baseline scores.

### Effects of Screen-Based 360° Contextual Simulation Videos on EEG Measures

#### Within-Group Analyses

In the experimental group, the correlation coefficients between the pretest and posttest values at electrode sites C3, Cz, and C4 were 0.550, 0.999, and 0.873, respectively. Two-tailed paired-sample *t* tests revealed statistically significant reductions in EEG power frequencies at all 3 electrode sites: C3 (*t*_27_=3.93; *P*<.001), Cz (*t*_27_=5.41; *P*<.001), and C4 (*t*_27_=4.82; *P*<.001). These findings indicate a significant decrease in EEG power frequencies after exposure to the screen-based 360° contextual simulation videos.

In the control group, the mean (SD) pretest and posttest EEG power frequencies at C3, Cz, and C4 were 13.47 (SD 11.85) Hz versus 11.50 (SD 9.59) Hz, 71.20 (SD 187.27) Hz versus 66.28 (SD 183.41) Hz, and 15.84 (SD 17.76) Hz versus 13.39 (SD 12.08) Hz, respectively. The correlation coefficients between the pretest and posttest values at C3, Cz, and C4 were 0.953, 0.999, and 0.803, respectively. Two-tailed paired-sample *t* tests for the control group yielded the following results: C3 (*t*_26_=1.79; *P*=.10), Cz (*t*_26_=2.05; *P*=.06), and C4 (*t*_26_=0.85; *P*=.41; Table 2). None of these *P* values reached statistical significance, thus indicating no significant changes in EEG power frequencies at any of the 3 electrode sites among participants who did not engage with the screen-based 360° contextual simulation videos.

#### Between-Group Analyses

The assumptions for between-group comparisons were assessed (details are provided in Multimedia Appendix 3). For the EEG power outcomes, the assumption of homogeneity of regression slopes was violated for C3; therefore, ANCOVA was not performed for C3. The ANCOVA results for Cz and C4 indicated significant between-group differences in posttest eyes-open resting-state EEG power after adjusting for baseline values (Table 2). Two-tailed independent-samples *t* tests revealed significant between-group differences in μSC at Cz and C4 (Table 3). In this study, μSC reflects eyes-open resting-state μ-band modulation rather than task-evoked suppression. These results indicate between-group differences in posttest resting-state μ-band indices at selected sites.

**Table 3.** Effects of the screen-based 360° contextual simulation videos on the μ-suppression scores between groups (n=55).

Variable μSC^a^	*t* test (df)	*P* value	Mean difference	SE difference	95% CI
C3	1.57 (53)	.13	16.89	10.77	−4.93 to 38.72
Cz	2.29 (53)	.03^b^	22.45	9.80	2.59 to 42.30
C4	2.21 (53)	.03^b^	23.69	10.71	1.20 to 45.38

a μSC: μ-band indices derived from eyes-open resting electroencephalography.

b *P*<.05. *P* values were calculated by performing 2-tailed independent samples *t* tests to examine the differences between groups.

## Discussion

### Principal Findings

This study revealed that exposure to screen-based 360° contextual simulation videos was associated with significant changes in both questionnaire outcomes and resting-state EEG indices. In the within-group analyses, the experimental group showed significant pretest-to-posttest improvements in SDS and EPQ scores and significant reductions in EEG power at C3, Cz, and C4, whereas the control group showed limited or nonsignificant changes in most measures. In between-group analyses, ANCOVA indicated that significant group effects for SDS, EPQ, and SCLS and resting-state EEG outcomes also differed between groups at selected electrode sites (Cz and C4), with additional between-group differences observed in the μ-rhythm indices at Cz and C4.

### Key Considerations

The findings indicate that the students positively responded to the use of screen-based 360° contextual simulation videos, reporting high levels of satisfaction with instructional design and teaching methodologies. These findings suggest that, compared with the control condition, exposure to screen-based 360° contextual simulation videos was associated with more favorable student-reported perceptions of simulation design and educational practices (SDS and EPQ scores) and higher self-reported satisfaction and self-confidence (SCLS scores). Additionally, the resting-state EEG indices (eyes-open) showed pre-post changes in the experimental condition, and group differences were observed at selected sensorimotor sites (eg, Cz and C4; Table 2). Because EEG data were collected in the eyes-open resting condition rather than during video viewing, these findings should be interpreted as overall pre-post sensorimotor rhythm modulation following learning exposure, not as direct video format-evoked action-observation responses. From an educational perspective, the use of screen-based 360° contextual simulation videos may represent a scalable approach to support procedural action observation in health professions training [5,6]. However, motor learning and cognitive engagement were not directly measured in this study and should be examined using objective performance outcomes and broader cognitive measures in future work.

EEG analyses indicated significant pre-post modulation of sensorimotor rhythm power at C3, Cz, and C4 in the eyes-open resting condition, with group differences at selected sites (eg, Cz and C4). Because EEG data were recorded in the eyes-open resting state at T0 and T1, these results reflect overall pre-post changes following exposure to the respective learning conditions rather than neural responses elicited during video viewing. Because the EEG analyses in this study focused on central sensorimotor sites (C3, Cz, and C4), we cannot attribute the observed effects to prefrontal regions or hemispheric dominance. In addition, μ-rhythm suppression is an indirect and nonspecific marker that can be influenced by attention, visual processing demands, and cognitive load. Therefore, these findings should be interpreted as EEG correlates consistent with sensorimotor rhythm modulation rather than as direct evidence of mirror neuron activation, cognitive engagement, or motor learning [8,11]. In future studies, EEG data should be recorded during video viewing under both conditions to examine whether the video format is associated with task-evoked sensorimotor EEG differences during procedural observation.

Taken together, the combination of higher posttest questionnaire scores (SDS, EPQ, and SCLS) and distinct sensorimotor EEG modulation (Cz, C4 power changes, and μ-rhythm indices) suggests that the screen-based 360° contextual simulation video condition was associated with more favorable learner perceptions and with pre-post modulation of resting sensorimotor EEG indices at selected sites. However, EEG data have limited spatial resolution, and μ-suppression is nonspecific [8]; due to the design of this study, specific neural generators could not be isolated, and the potential contributions of attention, visual processing, or unmeasured cognitive load could not be disentangled. Objective technical performance and longer-term retention were not assessed in this study and should be considered in future trials. Beyond EEG outcomes, students in the 360° video condition reported more favorable perceptions of simulation design and educational practices (SDS and EPQ scores) and higher satisfaction or self-confidence (SCLS scores) than did students in the control condition. These self-reported findings support the acceptability of screen-based 360° contextual simulation video learning as a supplement to undergraduate nursing skills education.

In interpreting these findings, Kolb [12] experiential learning cycle provides a useful lens, but the present intervention should be viewed as partially aligned with the model. The screen-based 360° contextual simulation videos primarily supported concrete experience (exposure to realistic procedural scenarios) and reflective observation (facilitated debriefing), and the linked procedural checklists may have supported abstract conceptualization. Consistent with evidence that observing clinical procedures can support skill retention [21], the intervention may help scaffold the early phases of learning. However, active experimentation (hands-on practice with feedback) was not implemented or objectively measured within the study sessions; thus, a complete experiential learning cycle cannot be claimed. In future work, hands-on performance should be integrated and assessed to operationalize active experimentation and retention more directly [22].

### Research Implications and Future Directions

From an educational perspective, screen-based 360° contextual simulation videos may offer a scalable supplement for undergraduate nursing skills education, particularly when supervised practice opportunities are constrained. In this study, the 360° video condition was associated with more favorable student-reported perceptions of simulation design and educational practices (SDS and EPQ scores) and higher satisfaction or self-confidence (SCLS scores), along with pre-post modulation of resting sensorimotor EEG indices at selected sites. These EEG findings should be interpreted as indirect, nonspecific neural correlates that are consistent with sensorimotor rhythm modulation rather than as direct evidence of mirror neuron activation, cognitive engagement, motor learning, or clinical competence [8,11]. In future studies, objective performance outcomes (eg, skills checklists), longer-term follow-up, and direct measures of cognitive load should be evaluated, and blended designs that integrate video-based observation with structured hands-on practice and feedback should be incorporated [23].

### Limitations

This study was conducted within an academic setting, which may have introduced an inherent power dynamic between the researchers and the participants. The presence of course instructors during data collection could have unintentionally influenced the students’ responses to the questionnaires, thereby introducing potential response bias and affecting the reliability of the self-reported measures. Additionally, the relatively small sample size and single-institution design may have limited the statistical power and generalizability of the findings, particularly in the context of diverse higher education environments. Because EEG data were collected in the eyes-open resting condition at T0 and T1 (and not during video viewing), the study could not test whether different video formats directly elicit different action-observation EEG responses. Because all participants completed the 4 modules in a fixed order and EEG data were collected only at baseline and immediately after the final (fourth) module, the EEG findings reflect an overall pre-post change across the full sequence rather than scenario-specific effects. Potential cumulative effects (eg, learning, fatigue, or changes in cognitive load) across the 4 modules cannot be disentangled. In addition, cognitive load was not directly measured, which limits the interpretability of the EEG indices. Although standardized proctoring procedures were used and confidentiality was emphasized, the presence of faculty members and teaching assistants during questionnaire completion may still have introduced social desirability or response bias, which may have affected the validity of the self-reported outcomes. Excluding questionnaire records with substantial missingness may have introduced response bias if missingness was not random (eg, systematic skipping among less engaged students). Although the number of excluded posttest records was small (3 records at T1), the questionnaire findings should be interpreted with caution in light of potential nonrandom missingness.

### Conclusions

This study suggests that integrating screen-based 360° contextual simulation videos into undergraduate nursing education is associated with improved learner-reported outcomes (SDS, EPQ, and SCLS scores) and modulation of eyes-open resting-state sensorimotor EEG indices. Future research should include analyses of objective performance outcomes and task-evoked EEG during video viewing to clarify relevant format-specific mechanisms.

## Supplementary material

10.2196/84720 Multimedia Appendix 1 Electroencephalography (EEG) data.

10.2196/84720 Multimedia Appendix 2 Study protocol.

10.2196/84720 Multimedia Appendix 3 Detailed assumption checks.

10.2196/84720 Checklist 1 CONSORT checklist.

## Abbreviations

AGFI: adjusted goodness-of-fit index
ANCOVA: analysis of covariance
CFI: comparative fit index
CONSORT: Consolidated Standards of Reporting Trials
EEG: electroencephalography
EPQ: Educational Practices Questionnaire
NFI: normed fit index
RMSEA: root mean square error of approximation
SCLS: Student Satisfaction and Self-Confidence in Learning Scale
SDS: Simulation Design Scale
TLI: Tucker-Lewis index
WRMR: weighted root mean square residual
