# Supplementary material for: 360° Contextual Simulation Videos for Undergraduate Nursing Students: Electroencephalography-Based Quasi-Experimental Study
Source: JMIR Nurs. 2026 Jun 30;9:e84720. doi: 10.2196/84720 (PMC13318206; doi:10.2196/84720)
Supplement: Multimedia Appendix 1 [file nursing-v9-e84720-s001.docx]

# Multimedia Appendix 1

EEG data were collected for both groups (experimental and control) at T0 and T1 using the same standardized protocol. Recordings were performed in the same setting and under the same eyes-open resting condition, with identical equipment, electrode placement (10–20 system), sampling parameters, and preprocessing procedures. Posttest EEG (T1) was obtained immediately after the fourth module in each group; the first 160 seconds of eyes-open resting EEG were extracted for analysis.

EEG recordings at both time points were conducted in a quiet room under standardized conditions (seated, minimal visual/auditory distractions). During the recordings, only the EEG physician and a trained research assistant were present to set up the equipment, monitor signal quality, and ensure participant safety; teaching staff were not involved in the EEG sessions. The same physician oversaw all the recordings and analyses to ensure procedural consistency and to minimize interassessor variability. To mitigate potential bias, EEG files were labelled using anonymized study codes, and preprocessing and feature extraction followed a prespecified standardized pipeline (e.g., fixed filter settings and extraction of the first 160 seconds of eyes-open resting EEG). EEG signals were recorded using a 19-electrode system following the international 10–20 standard. EEG assessments were conducted to record mirror neuron system brain wave activity (approximately 10 minutes) and anterior brain wave potential measurements (approximately 15 minutes), including filtering, artefact removal, referencing, and segmentation. Additionally, a cosine window was used to mitigate artefacts and improve signal quality. Importantly, μ-rhythm suppression is an indirect and nonspecific marker. It can be influenced by attention, visual processing demands, and cognitive load and therefore cannot be used to isolate or confirm mirror neuron activation. Accordingly, we interpret the observed changes in sensorimotor EEG power and μ-suppression as neural correlates consistent with action-observation–related processes rather than as direct evidence of mirror neuron activation or specific cognitive mechanisms. The μ-suppression score (μSC) was determined on the basis of the following formula: μSC = [−(µPbo – µPnbo)/µPnbo]×100, where μSC represents the percentage of μ suppression, μPbo indicates the μ power measured during the observation of simulated contextual videos, and μPnbo represents the μ power measured during the baseline (nonsimulated) video observation.
