# Supplementary material for: 360° Contextual Simulation Videos for Undergraduate Nursing Students: Electroencephalography-Based Quasi-Experimental Study
Source: JMIR Nurs. 2026 Jun 30;9:e84720. doi: 10.2196/84720 (PMC13318206; doi:10.2196/84720)
Supplement: Multimedia Appendix 2 [file nursing-v9-e84720-s002.docx]

**Multimedia Appendix 3. Study Protocol (Version 3.0; Date: [2024-5-23])**

**Study Title**

360° Contextual Simulation for Undergraduate Nursing Students: An Electroencephalography-Based Quasi-Experimental Study

**Objectives**

To evaluate whether integrating **screen-based 360° contextual simulation videos** into undergraduate nursing instruction is associated with changes in the following:

1. **Questionnaire-based learning outcomes**: Simulation Design Scale (SDS), Educational Practices Questionnaire (EPQ), and Student Satisfaction and Self-Confidence in Learning Scale (SCLS).
2. **EEG-based indices** recorded under an **eyes-open resting condition**, including the sensorimotor EEG power at **C3, Cz,** and **C4** and derived **μ-rhythm suppression indices**.

**Study Design**

Two-group, pretest–posttest quasiexperimental design with two time points:

- **T0 (baseline)**
- **T1 (immediately postintervention)**

**Setting**

A single university undergraduate nursing program; the study was conducted over **1 week (July 2024)**.

**Participants**

***Inclusion Criteria***

- Third-year undergraduate nursing students
- Able to communicate in Taiwanese, Hakka, Mandarin, or English
- Voluntary participation with informed consent

***Exclusion Criteria***

- Deafness or cognitive impairment based on available school health information and/or parental notification

**Group Allocation**

Participants were allocated to the **experimental** or **control** group using a **computer-generated allocation sequence**. Because recruitment was convenience-based within a single cohort and blinding was not implemented, the study is reported as quasiexperimental.

**Intervention and Control Conditions**

Both groups completed **four modules** in a **fixed, standardized order**. Module topics were identical across groups:

1. Collection of urine specimens from a urine drainage bag
2. Measurement and documentation of vital signs
3. Infant and paediatric CPR
4. Chest physiotherapy

***Experimental Condition (Screen-Based 360° Contextual Simulation Videos)***

- Delivery: University institutional e-learning platform via a web browser on a laptop; **no head-mounted display**
- “Interactivity”: learners can **pan/rotate** the viewing direction during observation
- Each module (~60 minutes total):
  - 360° video viewing: **~10–12 minutes**
  - Instructor-facilitated debriefing: **~20 minutes** using structured prompts (key steps, safety, clinical considerations)
  - Additional instructor guidance to connect observed actions to procedural steps/checklist and key concepts

***Control Condition (Dose-Matched Traditional Instruction with 2D Videos)***

- Delivery: Same institutional e-learning platform via a web browser on a laptop
- 2D videos: **self-developed by the same teaching team**; matched by topic and duration
- Each module (~60 minutes total):
  1. Didactic instruction + demonstration of key procedural steps
  2. 2D instructional video viewing: **~10–12 minutes**
  3. Instructor-facilitated discussion: **~20 minutes** using the same structured prompts
- No 360° video viewing; no high-fidelity mannequin simulation; no standardized patient

***Cognitive Load–Informed Design Considerations (Reported as Design Features)***

To minimize extraneous cognitive load during screen-based learning:

1. brief orientation on how to navigate videos before viewing
2. short videos focused on a single skill topic (~10–12 minutes)
3. standardized procedural steps reinforced via immediate structured debriefing
4. consistent viewing environment to reduce interface demands
   Cognitive load was **not directly measured**; these elements are reported as instructional design features.

**Procedures and Timeline**

- **Day 1 (T0):** baseline questionnaires + baseline EEG recording
- Completion of the four modules in a fixed order during the study week
- **Immediately after Module 4 (T1):** posttest questionnaires + posttest EEG recording

**Outcomes and Measures**

***Questionnaire Measures (T0 and T1)***

- SDS
- EPQ
- SCLS

***EEG Measures (T0 and T1)***

EEG recorded using standardized procedures; analyses focused on the following:

- Sensorimotor EEG power at **C3, Cz,** and **C4**
- μ-rhythm suppression indices derived from the resting EEG segment (details below)

**EEG Recording and Data Segment for Analysis**

At each time point (T0 and T1), EEGs were recorded in an **eyes-open resting condition**. The participants **did not perform an active task** during the analysis segment. For analysis, we extracted a continuous **160-second eyes-open** EEG segment from the recording (sampling rate of **500 Hz**). The extracted segment was used for spectral estimation and subsequent derivation of indices at **C3, Cz,** and **C4**.

**Sample Size Determination**

A priori power estimation was conducted using **G*Power** with assumptions aligned to the primary between-group comparison approach (ANCOVA adjusted for baseline). The minimum sample size requirement was **34** participants (details of the effect size/alpha/power settings are reported in the Methods section).

**Statistical Analysis Plan**

- Descriptive statistics: mean (SD)
- Within-group change: paired-samples t tests
- Between-group comparisons: ANCOVA (posttest as the dependent variable; group as the fixed factor; baseline as the covariate) when assumptions were met
- EEG power: within-group paired t tests; between-group ANCOVA for electrodes meeting the homogeneity of regression slopes assumption
- μ-suppression indices: independent-samples t tests
- Two-tailed tests; significance level P<.05; effect size partial η² where applicable

**Handling Missing Data**

Questionnaires with **≥10 missing item responses** were excluded from analysis. At T0, **0** questionnaires met the exclusion criteria; at T1, **3** questionnaires were excluded (experimental: **1**; control: **2**). The final analytic sample remained at **N=55** for the primary analyses.

**Ethics**

This study was approved by the IRB of MacKay Memorial Hospital (No. 21MMHIS438e). Informed consent was obtained from all the participants.
