# Supplementary material for: 360° Contextual Simulation Videos for Undergraduate Nursing Students: Electroencephalography-Based Quasi-Experimental Study
Source: JMIR Nurs. 2026 Jun 30;9:e84720. doi: 10.2196/84720 (PMC13318206; doi:10.2196/84720)
Supplement: Multimedia Appendix 3 [file nursing-v9-e84720-s003.docx]

# Multimedia Appendix 2

**Detailed Assumption Checks**

This appendix provides the detailed outputs of the assumption checks (Levene tests and homogeneity of regression slopes) and covariate effects used to justify the ANCOVA and between-group comparisons for the questionnaire outcomes and EEG outcomes.

**Effects of 360° Contextual Simulation Videos on Questionnaire Measures: Between-Group Analyses**

A covariate analysis revealed that the Levene’s variance homogeneity tests for the SDS, EPQ, and SCLS scores yielded the following results: *F*(1,38) = 1.56, *P*=.22; *F*(1,38) = .28, *P*=.60; and *F*(1,38) = 3.55, *P*=.07, respectively. None of these p values indicated statistical significance, thus confirming that the distributions of the variance in the SDS, EPQ, and SCLS scores were homogeneous across groups.

Furthermore, homogeneity of variance tests were performed to obtain regression slopes within groups, yielding the following interaction results between the independent variable (360° contextual simulation videos) and the covariates (baseline scores) for the SDS, EPQ, and SCLS: *F*(1,36) = 1.57, *P*=.22; *F*(1,36) = 2.69, *P*=.11; and *F*(1,36) = 2.70, *P*=.11, respectively. None of these p values indicated statistical significance, thus confirming that the linear relationships between the covariates and the independent variables were consistent across groups; accordingly, an ANCOVA was conducted.

Next, the effects of the covariates on the SDS, EPQ, and SCLS scores were assessed, and the ANCOVA results were as follows: *F*(1,35) = .07, *P*=.79 for the SDS; *F*(1,35) = 4.24, *P*=.047 for the EPQ; and *F*(1,35) = .58, *P*=.45 for the SCLS. The EPQ results were statistically significant, thus suggesting that the pretest EPQ variable significantly influenced the posttest variance. However, the pretest SDS and SCLS variables did not significantly affect the posttest scores. Nevertheless, the purpose of ANCOVA is to control for covariate influences, reduce error variance, and adjust mean differences; thus, the findings have practical significance.

ANCOVA further revealed a statistically significant between-group effect for all three measures: the SDS (*F*(1,35) = 7.80, *P*=.008), EPQ (*F*(1,35) = 6.00, *P*=.019), and SCLS (*F*(1,35) = 7.01, *P*=.012). These findings indicate that exposure to the 360° contextual simulation videos significantly affected the SDS, EPQ, and SCLS scores. The effect size values (η²) were .182 for the SDS, .146 for the EPQ, and .167 for the SCLS, thus highlighting the strong explanatory power of the intervention according to Cohen’s classification [20].

### Effects of 360° Contextual Simulation Videos on EEG Measures: Between-Group Analyses

Levene’s variance homogeneity tests for the EEG power at C3, C_Z_, and C4 yielded the following results: *F*(1,38) = 0.39, *P*=.54; *F*(1,38) = 0.02, *P*=.89; and *F*(1,38) = 1.46, *P*=.24, respectively. None of these p values indicate statistical significance, thus confirming that data dispersion between the two groups did not differ significantly for any of the electrodes, thereby meeting the homogeneity assumption.

Additionally, homogeneity of variance tests were performed to obtain regression slopes within groups regarding the interaction between the independent variable (360° contextual simulation videos) and the covariates (baseline EEG measures), yielding the following results: *F*(1,36) = 18.01, *P*<.001 for C3; *F*(1,36) = 0.09, *P*=.77 for C_Z_; and *F*(1,36) = 1.93, *P*=.17 for C4. The results pertaining to C3 were statistically significant, thus indicating that the linear relationships between the covariates and the independent variables were inconsistent across groups; accordingly, the homogeneity assumption was violated. Consequently, no ANCOVA was conducted for C3. However, the p values for C_Z_ and C4 did not indicate statistical significance, thus confirming that the regression slopes were homogeneous; accordingly, an ANCOVA was conducted for CZ and C4.

With respect to the effects of the covariates for C_Z_ and C4, the ANCOVA results were *F*(1,35) = 15007.60, *P*<.001 for C_Z_ and *F*(1,35) = 83.11, *P*<.001 for C4. Both results were statistically significant, thus indicating that the inclusion of pretest EEG measures for C_Z_ and C4 effectively controlled the analysis of variance and significantly influenced the posttest scores.

Between-group comparisons revealed statistically significant effects for C_Z_ (*F*(1,35) = 425.29, *P*<.001) and C4 (*F*(1,35) = 5.45, *P*=.026), thus indicating that exposure to 360° contextual simulation videos significantly affected EEG power at C_Z_ and C4 among participants in the experimental group. The effect size (η²) was .926 for C_Z_ and .138 for C4, indicating the strong explanatory power of the intervention, particularly at C_Z_, according to Cohen’s classification (Table 2).

In addition, independent-sample t tests were performed to assess the μ-suppression scores (μSCs) at C3, C_Z_, and C4 for the experimental and control groups, with values of 27.47 ± 35.76 vs. 10.58 ± 20.90, 38.26 ± 31.69 vs. 15.81 ± 21.75, and 32.51 ± 32.87 vs. 8.82 ± 28.49, respectively. Levene’s variance homogeneity tests yielded the following results: *F*(1,38) = 2.18, *P*=.15 for C3; *F*(1,38) = 2.09, *P*=.16 for C_Z_; and *F*(1,38) = 0.94, *P*=.34 for C4. None of these results indicate statistical significance, thus suggesting that the μSC distributions did not significantly differ between the groups.

The t test results were *t*(38) = 1.57, *P*=.13 for C3; *t*(38) = 2.29, *P*=.03 for C_Z_; and *t*(38) = 2.21, *P*=.03 for C4 (Table 3). The μSC value at C3 did not significantly differ between the experimental and control groups. However, significant differences in the μSC values were observed at C_Z_ and C4, thus indicating that the 360° contextual simulation videos influenced μ-suppression at these sites.
